# Innovation in organizational and educational strategies for undergraduate/graduate nursing: a qualitative approach

**DOI:** 10.1590/0034-7167-2024-0278

**Published:** 2025-04-28

**Authors:** Marcelle Miranda da Silva, Thayna de Assis Barros S. Thiago, Cristina Rosa Soares Lavareda Baixinho, Paulo Jorge Marcos Cruchinho, Bárbara Menezes Couto de Oliveira Santi Rossi, Ítalo Rodolfo Silva, Juliana Faria Campos

**Affiliations:** I Universidade Federal do Rio de Janeiro. Rio de Janeiro, Rio de Janeiro, Brazil; II Centro de Investigação, Inovação e Desenvolvimento em Enfermagem de Lisboa. Lisboa, Portugal

**Keywords:** Education, Nursing, Creativity, Diffusion of Innovation, Knowledge Management, Translational Science, Biomedical, Educación en Enfermería, Creatividad, Difusión de Innovaciones, Gestión del Conocimiento, Ciencia Traslacional Biomédica

## Abstract

**Objectives:**

to analyze the approach to innovation in organizational and educational strategies for undergraduate and graduate nursing from the perspective of students.

**Methods:**

this qualitative, descriptive study was conducted with 19 participants, including scientific initiation students, Stricto Sensu graduate students, and alumni from a single academic postgraduate program in Rio de Janeiro. Semi-structured interviews were conducted, and thematic analysis was performed while adhering to ethical guidelines.

**Results:**

three categories emerged: Curriculum Design and Teaching and Research Techniques; Teaching Evaluation and Learning Environment; and Culture of Innovation and Expected Outcomes. These categories encompassed strategies aimed at driving innovation, with a focus on curricular and teaching method changes to foster critical thinking, problem-solving skills, and evidence-based practice.

**Final Considerations:**

highlighted strategies include interdisciplinary training, the utilization of innovative tools, catalysts for knowledge translation, faculty training, and student engagement within the innovation ecosystem.

## RESUMEN

**Objetivos:**

analizar el enfoque de la innovación en las estrategias organizacionales y de enseñanza de pregrado y posgrado en enfermería desde la perspectiva de los estudiantes.

**Métodos:**

estudio cualitativo, descriptivo, desarrollado con 19 participantes, incluyendo estudiantes de iniciación científica, de posgrado Stricto Sensu y egresados de un mismo programa de posgrado académico en Río de Janeiro. Se realizaron entrevistas semiestructuradas y un análisis temático, respetando los aspectos éticos.

**Resultados:**

se desarrollaron tres categorías: Diseño curricular y técnicas de enseñanza e investigación; Evaluación de la enseñanza y del entorno de aprendizaje; Cultura de innovación y resultados esperados. Estas categorías abarcaron estrategias para impulsar la innovación, con énfasis en cambios en los currículos y en las técnicas de enseñanza, buscando fomentar el pensamiento crítico, las competencias para la resolución de problemas y la práctica basada en evidencia.

**Consideraciones Finales:**

las estrategias destacadas incluyen la formación interdisciplinaria, el uso de herramientas de innovación, catalizadores para la traducción del conocimiento, la capacitación docente y la participación de los estudiantes en el ecosistema de innovación.

## INTRODUCTION

In recent years, there has been a substantial effort worldwide to enhance knowledge translation, innovation, and cooperation between higher education institutions and industry^(1)^. In this context, transforming traditional higher education institutions into entrepreneurship-oriented institutions is essential, enabling them to innovate, recognize and create opportunities, work collaboratively, and take risks while responding independently to challenges^(2)^.

Innovation can have different meanings across various disciplines. In nursing, person-centered innovation involves the development and advancement of new systems, products, and processes to address urgent problems using rigorous methods and creativity, encompassing clinical, social, technological, educational, and process innovation^(3,4)^.

Innovation is crucial for improving the quality of nursing care and patient safety^(5)^. It encompasses the development of eight behavioral dimensions: 1) opportunity exploration; 2) idea generation; 3) idea research; 4) idea communication; 5) idea promotion; 6) idea advocacy; 7) idea implementation; and 8) overcoming obstacles^(6)^. Moreover, it requires interdisciplinary collaboration between nurses and other fields, such as engineering^(7)^.

Some nursing schools in North America and Asia have implemented organizational and educational strategies to align their curricula with international scientific standards, reflecting the rapid evolution of challenges and technologies. Enhancing the ability of nurses to think creatively and innovatively when addressing these challenges translates into results that can improve quality of life and achieve international goals for sustainable development^(8-10)^.

Such initiatives align with international nursing education standards, as outlined in the 2019-2023 strategic plan of the International Council of Nurses^(11)^, which emphasizes innovation, creativity, problem-solving, and transforming reality. However, on a global scale, gaps still exist in nursing curricula, as they do not include specific goals to operationalize this plan or facilitate knowledge translation and promote evidence-based practice^(12,13)^.

Although nurses demonstrate problem-solving and analytical skills, promoting innovation in education continues to face challenges, such as the predominance of traditional teaching models, which lead to low stimulation of creativity and innovative ideas^(9)^. Nursing education, from undergraduate studies to Stricto Sensu graduate programs, must promote creativity and the development of investigative and innovative competencies^(14,15)^. According to the Future for Nursing 2020-2030 report, innovation should be considered a core competency in nursing education and should be broadly incorporated into curricula^(4)^.

Thus, the question arises: how is innovation addressed in organizational and educational strategies for undergraduate and graduate nursing education?

## OBJECTIVES

To analyze the approach to innovation in organizational and educational strategies for undergraduate and graduate nursing from the perspective of students.

## METHODS

### Ethical Aspects

The study adhered to national and international ethical guidelines and was approved by the Research Ethics Committee of the Anna Nery School of Nursing, with the approval document attached to this submission. Written informed consent was obtained from all participants. The interviews were identified using the letter “P” followed by the interview number to ensure anonymity.

### Study Type

This qualitative, descriptive study followed the guidelines of the Consolidated Criteria for Reporting Qualitative Research (COREQ).

### Study Setting

The study was conducted within two research groups from a Stricto Sensu Academic Postgraduate Program in Nursing, located in Rio de Janeiro, Brazil. These research groups focus on integrating the development and incorporation of technologies and innovations into nursing and health care, teaching, and management practices. The themes of innovation and technology development have been extensively discussed within these groups, and all members were familiar with the topic.

The two groups consisted of a total of 41 members, including four faculty members, seven doctoral students, nine master’s students, nine alumni from the postgraduate program, six undergraduate scholarship students, and six voluntary scientific initiation students. Participants were conveniently invited for semi-structured interviews, ensuring representation across different student categories (undergraduate and postgraduate). The interview guide included the following primary question: “What strategies in nursing education (undergraduate and postgraduate) can facilitate addressing practical problems and foster creative thinking for innovative solutions?” Whenever appropriate, participants were asked to provide examples and personal experiences to deepen the understanding of the phenomenon. Notes were taken during the interviews to guide further exploration based on the examples and narratives shared.

Invitations to participate in the study were sent individually via WhatsApp^®^, using contact information obtained from pre-existing WhatsApp^®^ groups in which the lead researcher was a member. After confirming participation, messages were exchanged to schedule the date and time for the interviews. Individual interviews were conducted in August 2023 by a faculty member with a doctoral degree in nursing and who leads one of the groups. The interviews were conducted virtually on the Jitsi Meet^®^ platform, with only the audio being recorded.

The inclusion criteria were: membership in one of the research groups; enrollment as an undergraduate nursing student, with or without a scientific initiation scholarship; or being a regularly enrolled or alumni master’s/doctoral student. Exclusion criteria included participants who had been absent from the last two group meetings, which are held monthly, as well as faculty members. Faculty were excluded to maintain the study’s focus on capturing the experiences of the students, providing more specific insights into the approach to innovation, given that faculty perspectives may differ due to their role as methodology implementers.

After receiving individual invitations, no potential participant reported being unable to participate due to vacations, leave, or any other reason. The interviews lasted an average of 12 minutes, were transcribed verbatim, and presented to the participants, who did not suggest any changes or corrections. No interviews required repetition. During a joint meeting with both groups, participants provided positive feedback on the study’s findings.

### Data Analysis

The data were organized manually using Microsoft Word^®^, and coded by two authors who applied thematic content analysis^(16)^. Data saturation was discussed by the authors, based on the conceptual model of theoretical saturation^(17)^ and observations from field notes taken during the interviews.

The analysis followed the stages of pre-analysis, exploration of the material, and treatment of the results, with inference and interpretation. During the pre-analysis stage, recurring themes and concepts were identified to organize and prepare the data for the next step, which involved exploring the material to group it into relevant categories based on relationships between themes/concepts.

Finally, the data were interpreted by relating the findings to existing literature, providing a theoretical foundation for the study and making inferences based on the identified categories to address new knowledge, considering the context and implications of the findings.

## RESULTS

Out of the 37 group members, excluding faculty, eight were further excluded due to irregular attendance, as per the established criteria. Of the 19 interviewed members, four were undergraduate scientific initiation scholarship students, three were voluntary scientific initiation students, four were master’s students, five were doctoral students, and three were nurses who had graduated from the postgraduate program. Three categories were derived from the data (Chart 1).

**Chart 1:** Categories and Main Inferences, Rio de Janeiro, Brazil, 2023

Categories	Main Inferences
1: Curriculum Design and Teaching and Research Techniques	- Incorporate creativity, innovation, and entrepreneurship into core curricula. - Foster critical thinking. - Utilize active methodologies. - Expand practical opportunities, experiential learning, and challenge-based learning. - Teach students to conceptualize, design, and develop innovations for practical needs. - Integrate interdisciplinary teaching to enhance collaborative interactions. - Encourage interest in postgraduate studies.
2: Teaching Evaluation and Learning Environment	- Create authentic learning environments; - Engage students in research from the undergraduate level and facilitate knowledge translation; - Enable the assessment of nursing students’ creative capacity.
3: Culture of Innovation and Expected Outcomes	- Develop a shared understanding of innovation; - Value science in education and professional practice; - Create a structure to support innovation, including faculty training and development.

In Category 1, participants emphasized that nursing education should incorporate teaching strategies to enhance nurses’ ability to recognize problems, address them critically and creatively, seek solutions, and integrate knowledge:

*In practice, we encounter situations that challenge us and place nurses and their teams under tension. A good strategy would be the continuous implementation of critical and objective-focused thinking and reasoning. Often, we are not trained to transform adverse situations into solutions.* (P2)

*Active methodologies, such as simulations, can facilitate learning in nursing practice because they allow students to deepen their knowledge, discuss, reflect, and experience real situations in safe and controlled environments.* (P15)

*Pre-packaged responses should be avoided; pedagogical strategies that foster and stimulate debate around problem situations are necessary, which can be done with students from other disciplines.* (P7)

*Integration with graduate studies can help undergraduate students develop critical thinking by combining theoretical and practical knowledge in a specific manner.* (P19)

In Category 2, the data highlighted the need for educational policy reforms focusing on curriculum changes, with an emphasis on creating authentic learning environments:

*I come from a time when teaching relied on chalk and talk, with rare exceptions when a teacher required seminar presentations, as the best way to learn is by teaching others through an inverted classroom format. Today, things are different; nurses need a broader worldview, recognizing that it is ineffective to care for a patient who returns to the hostile environment that made them ill. The international context demands that nursing schools become more integrated with global issues and commit to innovative education and practices.* (P12)

In relation to the culture of innovation, in Category 3, P15 raised concerns related to faculty training, aligning with evaluations and learning environments discussed in Category 2:

*Faculty training is essential to foster student protagonism, scientific curiosity, and creative problem-solving, which can significantly contribute to initiating research projects, ultimately leading students to pursue postgraduate studies.* (P15)

Also within Category 3, participants highlighted innovation as a critical value and competency:

*Learning in healthcare is complex, as nurse training must go beyond reproducing techniques and knowledge, given that such knowledge may sometimes be ineffective in contexts different from those in which it was developed. I have always believed in the inventiveness of nursing, which daily demonstrates its adaptability. Necessity is the mother of invention, and that is how innovative solutions arise.* (P10)

Therefore, it is crucial to develop curricula aligned with contextual needs by incorporating approaches that promote innovation and enhance student learning.

## DISCUSSION

Investing in creativity and innovation is essential to keep pace with evolving health needs and overcome challenges for the sustainable functioning of healthcare systems^(18)^. Our results emphasize the responsibility of applying methods and techniques that foster innovation in nursing education, enabling nurses to act proactively in the face of challenges.

Proactive attitudes transform the perception of real-world problems and their solutions, fostering the development of nurses for advanced practice, leadership, and political advocacy^(10)^. This type of education inspires curiosity, values research and teaching, and includes the role of critical readers of the best evidence and preceptors who assist in developing new nurses in practice, elevating nursing to a science that transforms reality through knowledge application.

Creative and innovative thinking should underpin all core nursing disciplines, including their philosophical, epistemological, and methodological foundations, through the application of different strategies, as evidenced across the three categories. An interdisciplinary perspective is crucial, given the complexity of real-life situations and how they present themselves, involving interrelated issues that continuously reinforce one another^(19,20)^.

However, nursing education focused on innovation through teamwork is still in its infancy. Despite progress, corrective and reactive educational initiatives continue to dominate over reflective and proactive transformations that give meaning to experiences, as seen in metacognition^(21)^.

Interdependence in problem-solving is the foundation of teamwork; therefore, students and future professionals must be trained to work collaboratively and rely on each other to complete tasks. This involves achieving congruence in plans at the intersection of collaborative problems, ultimately leading to quality care^(22)^. Ensuring care quality is closely linked to promoting interdisciplinary education, combining knowledge and practices from different disciplines to enhance professional collaboration and generate new ideas^(23)^.

Studies conducted in China and the United States have highlighted the potential of integrating nursing with design, engineering, or industrial design, focusing on developing prototypes, patentable health products, and faculty training to enhance creative teaching skills and self-efficacy in teaching creativity^(22,24)^.

In China, interdisciplinary teaching was incorporated into nursing in 2016. Based on the need for creative and innovative training, part of the nursing curriculum in Taiwan emphasizes learning to identify essential health products, developed through innovative tools that facilitate identifying needs and selecting the best ideas for technological development^(25)^.

The importance of a culture that fosters entrepreneurship should be emphasized, as it, combined with interventions that enhance students’ entrepreneurial self-efficacy, can improve their entrepreneurial intentions. This requires project-based entrepreneurial classes, idea competitions, mentoring, funding opportunities, team formation, interdisciplinary teaching, among other aspects^(26)^. However, this was the weakest inference in our results, indicating that this mindset is not yet deeply ingrained. We need to broaden our vision to consider interdisciplinary strategies in teaching, research, and practice.

The wide range of information on the same situation or target audience, provided by teamwork, is fundamental for addressing problems and creating innovative solutions. Our data suggest, albeit tentatively, that professional education aimed at generating health-related products depends on enhancing this interdisciplinary mindset. In addition, it is necessary to advance nursing education in its generic capacities, encompassing skills for social and professional sustainability. These skills, which are primarily social, are underdeveloped in curricula and include creative thinking, self-directed learning, problem-solving, adaptability, communication skills, interpersonal skills, and teamwork^(4,27,28)^.

Among the strategies for developing these skills, with a focus on generating innovative ideas, the literature highlights the use of innovation management tools, such as brainstorming, design thinking, and experiential learning theory, among others^(4)^. For example, the application of brainstorming increases motivation and encourages teamwork^(27)^.

Professional education in postgraduate courses aims to support learning and creativity development, particularly based on research problems relevant to the diverse empirical realities faced by nurses. However, it is necessary to implement flexible, adaptable learning strategies aligned with the best evidence and focused on major challenges^(15)^.

Research projects, especially at the doctoral level, must be rigorously designed to conceive, develop, and implement innovative knowledge in interdisciplinary and complex contexts. The importance of securing project funding, particularly through nontraditional means, should be emphasized. This includes not only internal resources but also increased engagement in interprofessional collaboration and less reliance on independent researchers^(26,29,30)^.

We reiterate the importance of active student participation, starting at the undergraduate level, in research projects and/or the implementation of science. This approach has the potential to support development in various domains, from knowledge acquisition and competency building to the establishment of new attitudes that foster a culture of inquiry and behavioral change, ultimately enhancing the translation of knowledge into practice^(31)^.

A nursing student who neglects the potential of creative thinking and innovation, believing that existing care is sufficient, may pay less attention to individual patient needs and contribute minimally to shared decision-making. Conversely, a nursing student committed to seeking new solutions may better address the personal lives and needs of their patients, increasing the success of interventions^(32)^.

### Study limitations

We understand that all elements comprising the categories are dynamic and may vary according to creativity; there was no intention to cover every possible scenario. Concerning teaching and research strategies, one limitation was the inclusion of only undergraduate students with research experience, as well as students and graduates from an academic postgraduate program. Convenience sampling also posed limitations, as participants were selected based on their availability and closer proximity to the activities proposed by the research groups, which may not represent the complete diversity of the phenomenon of interest and does not allow for the generalization of the results.

There was also a limitation due to the restriction to the nursing field, despite the disciplinary focus of the study, as interdisciplinarity is essential for devising innovative solutions. Moreover, it is important to highlight a potential bias arising from the interviewer’s characteristics, who holds a leadership position in one of the research groups and serves as a faculty member. She endeavored to ensure objectivity based on her extensive experience in qualitative studies, using the guiding question, maintaining neutrality during interviews, and remaining aware of potential influences stemming from the participants’ awareness of her interests in the study topic. In light of these considerations, further studies should account for all these limitations.

### Contributions to the Field

While creativity should be encouraged from childhood, in professional education it can be further refined, enhancing value judgments as well as divergent (creative) and convergent (critical) thinking among peers and members of other disciplines, fostering the exploration of opportunities and the defense of ideas^(33)^.

To assess the innovative capacity of nursing students, research conducted in China reached a consensus that this capacity should be evaluated based on three indicators: creative spirit, innovation capacity, and innovation outcomes. Results should be measured through the application of knowledge during the educational process, even within simulated environments^(18)^.

Thus, addressing the problem to be solved is as crucial as devising ways to sustain innovation, without overlooking the influence of current and future economic, social, and political contexts, given the dynamic nature of reality and emerging and persistent health issues^(4)^.

This study clearly demonstrates that nursing schools have a dual role in fostering creativity and innovative knowledge, promoting curriculum restructuring and organizational changes, while also engaging researchers to create incentives that allow students to explore the challenges inherent in knowledge and evidence.

As an example, and based on literature, advanced nursing practice, including the Doctor of Nursing Practice (DNP), remains in its early stages of development in Brazil and requires ongoing encouragement. This practice aims to lead quality improvement initiatives within healthcare organizations, accelerating knowledge implementation^(34)^.

In general, providing opportunities for doctoral education as early as possible, whether in academic or practice settings, is a key strategy for enabling researchers to dedicate more of their careers to producing and applying knowledge, thereby expanding educational opportunities for nurses in practice settings. The growth of nursing as a science is closely tied to the involvement of students and professionals in research, with a commitment to applying it based on scientific evidence, as research is an inseparable component of education and professional practice^(34,35)^.

## FINAL CONSIDERATIONS

To address innovation in undergraduate and postgraduate nursing education, it is crucial to restructure curricula, teaching strategies, and research methods to develop creative solutions that meet contextual needs, thereby enhancing nursing’s ability to ensure population health and well-being. Recognizing that the approach to certain problems requires an interdisciplinary and multisectoral perspective is key.

This necessitates the continuous evaluation of the teaching-learning process, involving the application of innovative methods that go beyond traditional examinations. These methods include oral presentations, simulations, case studies, among others. In this context, it is essential to value student feedback, allowing them to adjust their learning as needed, in alignment with the competencies being assessed.

On a broader scale, within organizational strategies, efforts should focus on fostering a culture of innovation. This includes preparing environments with adequate resources, both in terms of materials and physical spaces, and ensuring faculty development. Through this approach, innovation can drive improvements in practice, teaching, and nursing and health management, contributing to greater visibility for the nursing profession and increased job satisfaction for nurses.
