# Supplementary material for: Innovation in organizational and educational strategies for undergraduate/graduate nursing: a qualitative approach
Source: Rev Bras Enferm. 2025 Apr 28;78(2):e20240278. doi: 10.1590/0034-7167-2024-0278 (PMC12037188; doi:10.1590/0034-7167-2024-0278)
Supplement: Translation

# Translation: Inovação nas estratégias organizacionais e de ensino de graduação/pós-graduação em enfermagem: uma abordagem qualitativa

**DOI:** 10.1590/0034-7167-2024-0278pt

**Authors:** Marcelle Miranda da Silva, Thayna de Assis Barros S. Thiago, Cristina Rosa Soares Lavareda Baixinho, Paulo Jorge Marcos Cruchinho, Bárbara Menezes Couto de Oliveira Santi Rossi, Ítalo Rodolfo Silva, Juliana Faria Campos

**Affiliations:** I Universidade Federal do Rio de Janeiro. Rio de Janeiro, Rio de Janeiro, Brasil; II Centro de Investigação, Inovação e Desenvolvimento em Enfermagem de Lisboa. Lisboa, Portugal

**Keywords:** Educação em Enfermagem, Criatividade, Difusão de Inovações, Gestão do Conhecimento, Tradução do Conhecimento

## Abstract

**Objetivos:**

analisar a abordagem da inovação nas estratégias organizacionais e de ensino de graduação e pós-graduação em enfermagem na perspectiva dos discentes.

**Métodos:**

estudo qualitativo, descritivo, desenvolvido com 19 participantes, incluindo alunos de iniciação científica, de pós-graduação Stricto Sensu e egressos de um mesmo programa de pós-graduação acadêmico no Rio de Janeiro. Foram aplicadas entrevistas semiestruturadas e realizada análise temática, respeitando-se os aspectos éticos.

**Resultados:**

três categorias foram desenvolvidas: Desenho do currículo e técnicas de ensino e pesquisa; Avaliação de ensino e ambiente de aprendizagem; Cultura de inovação e resultados esperados. Essas categorias englobaram estratégias para impulsionar a inovação, com ênfase em mudanças nos currículos e nas técnicas de ensino, visando fomentar o pensamento crítico, as competências de resolução de problemas e a prática baseada em evidências.

**Considerações Finais:**

as estratégias destacadas incluem: formação interdisciplinar, utilização de ferramentas de inovação, catalisadores para a tradução do conhecimento, capacitação docente e participação dos alunos no ecossistema de inovação.

## INTRODUÇÃO

Nos últimos anos, temos assistido a um esforço substancial em todos os países para incrementar conjuntamente a tradução do conhecimento, a inovação e a cooperação entre as instituições de ensino superior e a indústria^(1)^. Neste contexto, é fundamental transformar as instituições de ensino superior tradicionais em instituições orientadas para o empreendedorismo, de forma a inovar, reconhecer e criar oportunidades, trabalhar em equipe e assumir riscos, respondendo aos desafios por conta própria^(2)^.

A inovação pode ter diferentes significados para diferentes disciplinas. Na enfermagem, a inovação centrada na pessoa engloba a formulação e o avanço de novos sistemas, produtos e processos para solucionar problemas urgentes, com base em métodos rigorosos e na criatividade, abrangendo inovação clínica, social, tecnológica, educacional e de processo^(3,4)^.

A inovação é um elemento importante para a melhoria da qualidade dos cuidados de enfermagem e da segurança do paciente^(5)^. Ela compreende o desenvolvimento de um conjunto de oito dimensões comportamentais: 1) a exploração de oportunidades; 2) a geração de ideias; 3) a pesquisa de ideias; 4) a comunicação de ideias; 5) a promoção de ideias; 6) a defesa de ideias; 7) a aplicação de ideias; e 8) a superação de obstáculos^(6)^. Além disso, requer dos enfermeiros uma colaboração interdisciplinar com outras disciplinas, como a engenharia^(7)^.

Algumas escolas de enfermagem, em países da América do Norte e Ásia, têm implementado estratégias organizacionais e de ensino para adequar seus currículos aos padrões internacionais da ciência, que refletem a rápida evolução dos problemas e das tecnologias. Melhorar a competência do enfermeiro para pensar de forma criativa e inovadora na solução desses problemas significa gerar resultados capazes de melhorar a condição de vida das pessoas e alcançar as metas internacionais para o desenvolvimento sustentável^(8-10).^

Tais iniciativas estão em consonância com os padrões internacionais de educação em enfermagem, conforme disposto no plano estratégico 2019-2023 do Conselho Internacional de Enfermeiros^(11)^, o qual inclui a inovação, a criatividade, o foco na solução de problemas e na transformação da realidade. Entretanto, em uma perspectiva global, ainda se identificam lacunas nos currículos de enfermagem que não contemplam metas específicas para operacionalizar este plano, tampouco para facilitar a tradução do conhecimento e promover a prática baseada em evidências^(12,13)^.

Embora os enfermeiros demonstrem habilidades para análise e resolução de problemas, a promoção da inovação na educação ainda enfrenta desafios, como o predomínio do modelo tradicional de ensino, que resulta em baixa estimulação da criatividade e de ideias inovadoras^(9)^. A educação em enfermagem, desde a graduação e, especialmente, na pós-graduação *Stricto Sensu*, deve promover a criatividade e o desenvolvimento de competências investigativas e inovadoras^(14,15)^. De acordo com o relatório *Future for Nursing* 2020-2030, a inovação deve ser considerada uma competência a ser desenvolvida na educação em enfermagem e precisa ser incorporada ao currículo de forma ampliada^(4)^.

Desse modo, questiona-se: como a inovação é abordada nas estratégias organizacionais e de ensino de graduação e pós-graduação em enfermagem?

## OBJETIVOS

Analisar a abordagem da inovação nas estratégias organizacionais e de ensino de graduação e pós-graduação em enfermagem sob a perspectiva dos discentes.

## MÉTODOS

### Aspectos éticos

O estudo foi conduzido de acordo com diretrizes éticas nacionais e internacionais e aprovado pelo Comitê de Ética em Pesquisa da Escola de Enfermagem Anna Nery, cujo parecer está anexado à presente submissão. O Consentimento Livre e Esclarecido foi obtido de todos os participantes por meio escrito. Os depoimentos foram identificados com a letra “P” seguida do número da entrevista, de modo a garantir o anonimato.

### Tipo de estudo

Estudo qualitativo, descritivo, guiado pelas diretrizes do *Consolidated Criteria for Reporting Qualitative Research* (COREQ).

### Cenário do estudo

O estudo foi realizado no âmbito de dois grupos de pesquisa de um Programa Acadêmico de Pós-Graduação Stricto Sensu em Enfermagem, localizado no Rio de Janeiro, Brasil. Os grupos de pesquisa possuem linhas de atuação que integram a elaboração e incorporação de tecnologias e inovações nas ações de cuidado, ensino e gestão em enfermagem e saúde. As temáticas relacionadas à inovação e ao desenvolvimento de tecnologia têm sido amplamente discutidas nos grupos, e todos os seus integrantes possuíam familiaridade com o tema.

Os dois grupos contavam, ao todo, com 41 membros, sendo quatro docentes, sete doutorandos, nove mestrandos, nove egressos da pós-graduação, seis alunos de graduação bolsistas e seis voluntários de iniciação científica. Os participantes foram convidados de forma conveniente para a entrevista semiestruturada, com a preocupação de representar cada categoria de estudantes (graduação e pós-graduação). O roteiro de entrevista incluía a seguinte questão orientadora: “Quais estratégias na educação em enfermagem (graduação e pós-graduação) podem facilitar a abordagem dos problemas na prática e o pensamento criativo de soluções inovadoras?”. Sempre que apropriado, foram solicitados exemplos e relatos de experiências para aprofundar a compreensão do fenômeno. Algumas anotações foram realizadas durante as entrevistas para orientar o aprofundamento de questões conforme os exemplos e relatos apresentados.

O convite para participar do estudo foi enviado individualmente aos potenciais participantes pelo *WhatsApp* ^®^, utilizando contatos telefônicos obtidos em grupos de *WhatsApp* ^®^ preexistentes, dos quais a pesquisadora principal participa. Após o aceite, as mensagens foram utilizadas para agendar o dia e o horário das entrevistas. As entrevistas foram realizadas individualmente em agosto de 2023, conduzidas por uma docente, doutora em enfermagem e líder de um dos grupos, na sala virtual utilizada para as reuniões, na plataforma *Jitsi Meet* ^®^, sendo gravado apenas o áudio.

Os critérios de inclusão foram: integrar um dos grupos de pesquisa; ser aluno do curso de graduação em enfermagem com ou sem bolsa de iniciação científica; ser aluno regularmente matriculado ou egresso dos cursos de mestrado e/ou doutorado. Foram excluídos os participantes que estiveram ausentes nas duas últimas reuniões dos grupos, que acontecem mensalmente, e os docentes. A exclusão dos docentes foi realizada para evitar desvio do foco do objetivo, desenhado para captar exclusivamente a experiência dos discentes, proporcionando insights mais específicos sobre a abordagem da inovação, uma vez que os docentes podem ter perspectivas diferentes por serem os implementadores das metodologias.

Após o convite individual, nenhum potencial participante alegou impossibilidade de participar devido a férias, licença ou qualquer outro motivo. As entrevistas duraram, em média, 12 minutos, foram transcritas na íntegra e apresentadas aos participantes, que não fizeram comentários ou correções. Nenhuma entrevista precisou ser repetida. Em uma reunião conjunta dos grupos, os participantes forneceram um feedback positivo sobre os resultados.

### Análise dos dados

Os dados foram organizados manualmente no *Microsoft Word* ^®^, codificados por duas autoras, que utilizaram a análise de conteúdo com abordagem temática^(16)^. A saturação dos dados foi discutida pelas mesmas, com base no modelo conceitual da saturação teórica^(17)^ e nas observações das notas de campo feitas durante as entrevistas.

A análise seguiu as etapas de pré-análise, exploração do material, e tratamento dos resultados obtidos, com inferência e interpretação. Na pré-análise, foram identificados os temas e conceitos recorrentes para organizar e preparar os dados para a etapa seguinte, em que se realizou a exploração do material para agrupamento em categorias relevantes, de acordo com as relações entre os temas/conceitos. Por fim, a interpretação dos dados foi feita com base na relação dos achados com a literatura existente, proporcionando a fundamentação teórica do estudo, além de inferências a partir das categorias identificadas para abordar o novo conhecimento, considerando o contexto e as implicações dos achados.

## RESULTADOS

Dos 37 membros dos grupos, já excluídos os docentes, outros oito foram excluídos porque não estavam assíduos, conforme critério delimitado. Dos 19 membros entrevistados, quatro eram alunas bolsistas de iniciação científica, três eram alunas voluntárias de iniciação científica, quatro mestrandos, cinco doutorandos, e três enfermeiros egressos do programa de pós-graduação. Foram desenvolvidas três categorias derivadas dos dados (Quadro 1).

**Quadro 1:** Categorias e principais inferências, Rio de Janeiro, Brasil, 2023

Categorias	Principais Inferências
1. Desenho do currículo e técnicas de ensino e pesquisa	- Incorporar criatividade, inovação e empreendedorismo nos currículos fundamentais; - Estimular o pensamento crítico; - Utilizar metodologias ativas; - Ampliar as oportunidades práticas, a aprendizagem experimental e baseada em desafios; - Ensinar a conceituar, projetar e desenvolver uma inovação para uma necessidade da prática; - Integrar ensino interdisciplinar para melhorar as interações colaborativas; - Estimular o interesse pela pós-graduação.
2. Avaliação de ensino e ambiente de aprendizagem	- Criar ambientes de aprendizagem autênticos; - Envolver o estudante na pesquisa desde a graduação, e facilitar a tradução do conhecimento; - Possibilitar a avaliação da capacidade criativa dos estudantes de enfermagem.
3. Cultura de inovação e resultados esperados	- Desenvolver um significado compartilhado de inovação; - Valorizar a ciência na formação e na prática profissional; - Criar estrutura para apoiar a inovação, o que inclui a capacitação e o desenvolvimento do corpo docente.

Na categoria 1, os participantes declararam que a educação em enfermagem deve incluir estratégias de ensino para aumentar a capacidade do enfermeiro de reconhecer os problemas, tratá-los de forma crítica e criativa, buscando soluções e articulando conhecimentos:

*Na prática nos deparamos com situações que nos fazem pensar, e que deixam o enfermeiro e equipe em tensão. Uma boa estratégia seria sempre a implementação de um pensamento e raciocínio crítico voltado para a prática em objetivo. Muitas das vezes não somos educados a transformar situações adversas em solução.* (P2)

*As metodologias ativas, como simulações, podem facilitar o aprendizado das práticas de enfermagem, porque permitem que o aluno aprofunde, discuta, reflita e vivencie situações reais em ambientes seguros e controlados.* (P15)

*Deve-se evitar o recebimento de respostas prontas; são necessárias dinâmicas pedagógicas que criem e estimulem o debate acerca de uma situação-problema, o que pode ser feito com alunos de outras disciplinas.* (P7)

*A integração com a pós-graduação pode auxiliar os alunos de graduação no desenvolvimento do pensamento crítico, agregando conhecimentos teóricos e práticos de forma específica*. (P19)

Na categoria 2, os dados evidenciaram que a política educacional precisa de mudanças curriculares, com destaque para a necessidade de criar ambientes de aprendizagem autênticos:

*Sou de um tempo que o ensino era de cuspe e giz, com raras exceções quando algum professor exigia a apresentação de seminário, já que a melhor forma de apreender os conhecimentos é ter que ensiná-los, numa aula invertida. Hoje a história é outra, o enfermeiro necessita ter uma visão ampliada de mundo, ciente de que não adianta cuidar do paciente se o mesmo retorna para o ambiente hostil que o fez ficar doente. O cenário internacional vem exigindo que escolas de enfermagem se tornem mais integradas aos assuntos do mundo e comprometam-se com uma educação e práticas inovadoras*. (P12)

Sobre a cultura de inovação, na categoria 3, P15 trouxe questões relacionadas à capacitação docente, e que conversam com a avaliação e o ambiente de aprendizagem na categoria 2:

*É preciso capacitação docente para propiciar o protagonismo do aluno, o pensamento, a curiosidade científica, e a busca por soluções criativas, o que pode, inclusive, ter papel importante na iniciação de projetos de pesquisa, levando o estudante a uma futura busca pela pós-graduação.* (P15)

Ainda na categoria 3, os participantes apontam a inovação como valor, reconhecida como competência crítica:

*Aprender na área da saúde é complexo, porque a formação dos enfermeiros necessita ir além da reprodução das técnicas e conhecimentos, já que, às vezes, tais conhecimentos são ineficazes em contextos cujas características são diferentes daqueles onde os conceitos foram produzidos. Eu sempre acreditei na inventiva da enfermagem, que mostra diariamente capacidade de adaptação. Ou seja, a necessidade é que ensina o sapo a pular. Daí surgem soluções inovadoras.* (P10)

Desse modo, é fundamental desenvolver currículos alinhados com as necessidades dos contextos, a partir da incorporação de abordagens que promovam a inovação e aprimorem a aprendizagem dos estudantes.

## DISCUSSÃO

É preciso investir em criatividade e inovação para acompanhar a evolução das necessidades de saúde e superar os desafios para o funcionamento sustentável dos sistemas de saúde^(18)^. Nossos resultados destacam a responsabilidade de aplicar métodos e técnicas que estimulem a inovação no ensino de enfermagem, permitindo que o enfermeiro atue de forma proativa diante dos desafios.

Atitudes proativas transformam a percepção dos problemas da vida real e suas abordagens, promovendo a formação de enfermeiros para a prática avançada, liderança e ativismo político^(10)^. Essa educação, que motiva e desperta a curiosidade, valoriza a pesquisa e o ensino, incluindo o papel de leitores críticos das melhores evidências e de preceptores que auxiliam no desenvolvimento de novos enfermeiros na prática, elevando a enfermagem a uma ciência que transforma a realidade pela aplicação do conhecimento.

O pensamento criativo e inovador deve orientar todas as disciplinas básicas da enfermagem, suas bases filosóficas, epistemológicas e metodológicas, por meio da aplicação de diferentes estratégias, conforme evidenciado nas três categorias. É necessário adotar uma perspectiva interdisciplinar, dada a complexidade das situações e como elas se apresentam na vida real, envolvendo questões inter-relacionadas que se retroalimentam constantemente^(19,20)^.

No entanto, a educação em enfermagem com foco na inovação a partir do trabalho em equipe ainda é recente. Apesar dos avanços, ainda prevalecem iniciativas educacionais corretivas e reativas, em detrimento de transformações reflexivas e proativas que atribuam sentido às experiências, como na metacognição^(21)^.

A interdependência para a solução de problemas é a base do trabalho em equipe; assim, os estudantes, futuros profissionais, devem ser educados para agir em conjunto e depender uns dos outros para a conclusão dos trabalhos. Isso envolve buscar congruência nos planos na interseção dos problemas colaborativos, resultando em cuidados de qualidade^(22)^. Assegurar a qualidade do cuidado está fortemente relacionado à promoção da educação interdisciplinar, à conjunção de saberes e práticas de diferentes disciplinas para aumentar a colaboração entre profissionais e gerar novas ideias^(23)^.

Estudos realizados na China e nos Estados Unidos destacaram as potencialidades da integração entre enfermagem, design, engenharia ou desenho industrial, com foco na elaboração de protótipos, produtos de saúde patenteáveis e capacitação de docentes para reforçar habilidades de ensino criativo e a autoeficácia para ensinar criatividade^(22,24)^.

Na China, o ensino interdisciplinar foi incluído na enfermagem em 2016, e, com base na necessidade de formação criativa e inovadora, parte do currículo de enfermagem em Taiwan concentra-se no aprendizado de como identificar um produto necessário para a saúde, desenvolvido com a aplicação de ferramentas de inovação que facilitam a identificação de necessidades e a escolha da melhor ideia para construir uma tecnologia^(25)^.

Destaca-se a importância da cultura para o empreendedorismo, pois, associada ao desenvolvimento de intervenções que aumentem a autoeficácia empreendedora dos alunos, pode melhorar sua intenção empreendedora. Isso requer aulas baseadas em projetos empreendedores, concursos de ideias, mentorias, financiamentos, formação de equipes, ensino interdisciplinar, entre outros aspectos^(26)^. Entretanto, essa foi a inferência mais fraca em nossos resultados, o que sugere que essa mentalidade ainda não está suficientemente enraizada. Precisamos ampliar a visão para pensar em estratégias interdisciplinares no ensino, na pesquisa e na prática.

A ampla gama de informações sobre a mesma situação ou público-alvo, proporcionada pelo trabalho em equipe, é fundamental para abordar o problema e conceber soluções criativas. Nossos dados apontam, ainda que de forma incipiente, que a educação profissional para a geração de produtos voltados à saúde depende do aprimoramento desse pensamento interdisciplinar. Além dessa dependência, é necessário avançar na educação em enfermagem em suas capacidades genéricas, que englobem habilidades para a sustentabilidade social e profissional. Essas habilidades, essencialmente sociais, são pouco desenvolvidas nos currículos e incluem pensamento criativo, aprendizagem autogerida, resolução de problemas, adaptabilidade, habilidades de comunicação, habilidades interpessoais e trabalho em equipe^(4,27,28)^.

Entre as estratégias para desenvolver essas habilidades, com foco na geração de ideias inovadoras, destaca-se, na literatura, a aplicação de ferramentas de gestão da inovação, como o brainstorming, o *design thinking* e a teoria da aprendizagem experiencial, entre outras^(4)^. A aplicação do brainstorming, por exemplo, aumenta a motivação e incentiva o trabalho em equipe^(27)^.

A educação profissional nos cursos de pós-graduação busca apoiar o aprendizado e o desenvolvimento da criatividade, especialmente com base nos problemas de pesquisa relacionados às diversas realidades empíricas dos enfermeiros. No entanto, é necessário implementar estratégias de aprendizado que sejam flexíveis, adaptáveis, alinhadas às melhores evidências e focadas nos grandes desafios^(15)^.

Os projetos de pesquisa, especialmente no doutorado, precisam ser elaborados com rigor e bem projetados para conceber, desenvolver e implementar conhecimento inovador em contextos interdisciplinares e complexos. Ressalta-se a importância de os projetos serem financiados, especialmente de maneira não tradicional, ou seja, não apenas com recursos internos, mas com maior engajamento em colaboração interprofissional e menos conduzidos por pesquisadores independentes^(26,29,30)^.

Reiteramos a importância da participação ativa do aluno desde a graduação em projetos de pesquisa e/ou na implementação de ciência, considerando o potencial de seu desenvolvimento em diferentes domínios, desde o aprendizado de conhecimentos e competências até a definição de novas atitudes que fomentam a cultura de investigação e a mudança de comportamento que potencializa a tradução do conhecimento para a prática^(31)^.

O aluno de enfermagem que negligencia o potencial do pensamento criativo e da inovação, considerando que os cuidados existentes são suficientes, pode preocupar-se menos com a individualidade da pessoa e promover pouco ou nenhum compartilhamento na tomada de decisão. Por outro lado, o aluno de enfermagem disposto a buscar novas soluções pode prestar mais atenção à vida pessoal e às necessidades de seus pacientes, o que aumentará o sucesso da intervenção^(32)^.

### Limitações do estudo

Entendemos que todos os elementos que compuseram as categorias são dinâmicos e podem variar de acordo com a criatividade; não houve a intenção de discutir todas as possibilidades. Quanto às estratégias de ensino e pesquisa, uma limitação foi a inclusão de apenas alunos da graduação com experiência em pesquisa, bem como apenas alunos e egressos de um programa acadêmico de pós-graduação. A amostragem por conveniência também trouxe limitações, uma vez que os participantes foram selecionados com base em sua disponibilidade e maior proximidade com as atividades propostas pelos grupos de pesquisa, o que pode não representar a diversidade completa do fenômeno de interesse e não permitir a generalização dos resultados.

Houve também uma limitação quanto à restrição à área da enfermagem, apesar do enfoque disciplinar do estudo, pois a interdisciplinaridade é essencial para pensar em soluções inovadoras. Ademais, é relevante destacar um possível viés decorrente da característica da entrevistadora, que ocupa uma posição de liderança em um dos grupos de pesquisa e exerce atividade docente. Ela buscou garantir a objetividade com base em sua vasta experiência em estudos qualitativos, utilizando a questão orientadora, mantendo a neutralidade durante as entrevistas e se conscientizando das possíveis interferências decorrentes do conhecimento dos participantes sobre seus interesses no tema do estudo. Diante disso, recomenda-se a realização de novos estudos que levem em conta todas essas limitações.

### Contribuições para a Área

Embora a criatividade deva ser estimulada desde a infância, na educação profissional ela pode ser aprimorada, melhorando o juízo de valores, bem como os pensamentos divergente (criativo) e convergente (crítico) entre os colegas e membros de outras disciplinas, na exploração de oportunidades e na defesa de ideias^(33)^.

Para a avaliação da capacidade de inovação dos alunos de enfermagem, uma pesquisa realizada na China apresentou um consenso de que essa capacidade deve ser avaliada com base em três indicadores: espírito criativo, capacidade de inovação e resultados de inovação. Os resultados devem ser mensurados a partir da aplicação do conhecimento durante o processo educacional, mesmo que em ambiente simulado^(18)^.

Assim, abordar o problema a ser resolvido é tão importante quanto pensar em meios para sustentar a inovação, sem desconsiderar as influências dos contextos econômicos, sociais e políticos no presente e no futuro, considerando a dinâmica da realidade e os problemas de saúde novos, emergentes e persistentes^(4)^.

Este estudo deixa claro que as escolas de enfermagem têm um papel dual no impulso à criatividade e ao conhecimento inovador, promovendo a reestruturação curricular e mudanças organizacionais, além de envolverem os investigadores para criar incentivos que permitam aos alunos explorar os desafios inerentes ao conhecimento e à evidência.

A título de exemplo, e com base na literatura, destaca-se a prática avançada de enfermagem, incluindo o doutorado em prática, que ainda está em fase de desenvolvimento inicial no Brasil e precisa ser cada vez mais estimulado. Tal prática carrega fortemente o objetivo de liderar iniciativas de melhoria da qualidade para as organizações de saúde, acelerando a implementação do conhecimento^(34)^.

Em geral, oportunizar a educação de doutorado o mais precocemente possível, seja em nível acadêmico ou em prática, constitui uma estratégia importante para que o pesquisador possa dedicar mais tempo de sua carreira à produção e aplicação do conhecimento, expandindo a educação para enfermeiros nos cenários de prática. O crescimento da enfermagem enquanto ciência está relacionado ao envolvimento do aluno e do profissional na pesquisa, com o compromisso de aplicá-la com base nas evidências científicas, visto que a pesquisa é um componente indissociável da educação e da prática profissional^(34,35)^.

## CONSIDERAÇÕES FINAIS

Para abordar a inovação no ensino de graduação e pós-graduação em enfermagem, destaca-se a necessidade de reestruturar os currículos, as estratégias de ensino e os métodos de pesquisa, de modo a alcançar soluções criativas que atendam às necessidades dos contextos, promovendo a capacidade da enfermagem de assegurar o bem-estar em saúde da população, ciente de que a abordagem de certos problemas precisa ser interdisciplinar e multissetorial.

Para isso, é necessário recorrer à avaliação contínua do processo de ensino-aprendizagem, o que envolve a aplicação de métodos inovadores, que não se limitem a provas tradicionais, incluindo apresentações orais, simulações, estudos de casos, entre outros. Nesse contexto, é fundamental valorizar o feedback dos estudantes, permitindo-lhes ajustar seu aprendizado conforme necessário, em alinhamento com as competências a serem avaliadas.

Em um aspecto mais amplo, no âmbito das estratégias organizacionais, os dados abrangem a promoção de uma cultura de inovação, direcionando esforços para a preparação dos ambientes, incluindo a disponibilidade de recursos materiais e espaços físicos, além da capacitação docente. Assim, a inovação poderá gerar melhorias na prática, no ensino e na gestão em enfermagem e saúde, contribuindo para a visibilidade da enfermagem e a satisfação dos enfermeiros.
